# Literary Notes

**Published:** 1901-07

**Authors:** 


### LITERARY NOTES.

THE New York Pharmacal Association, Yonkers, N.Y., has issued a pamphlet entitled, Facts and Figures, medical and otherwise, from the census of 1900, and other reliable sources. It is a handsome brochure of 64 pages, containing maps, charts, tables and texts of great interest and which cannot be found elsewhere grouped between two covers. It will be supplied on application to the publishers.

---

Dr. S. A. ARANY, of Carlsbad, has published the second edition of the British-American guide to Carlsbad. It is a handsomely printed and well illustrated brochure of 90 pages. Price, 1 shilling, and may be obtained at any of Thomas Cook & Sons' offices.

---

J. B. LIPPINCOTT CO., publishers, Philadelphia, announces as in course of publication Dr. George Henry Fox's Atlas of Skin Diseases. It is issued in monthly parts, consisting of five colored life-like plates, reproduced photographically from actual cases. Each part also contains fourteen pages of text and five pages descriptive of the plates. The entire work when completed will embrace a comprehensive and practical treatise upon the subject of skin diseases by one of the highest American authorities; also eighty full-page plates executed in a faithful and superior manner. This work is of great value not only to dermatologists, but to general practitioners as well.

---

THE *Quarterly Journal of Inebriety* will publish in the July number a symposium of the most authoritative scientific papers, recently read before medical societies in this country and Europe, on the physiological and pathological action of alcohol. These papers will contain the latest facts and conclusions, on the action of alcohol as a beverage and medicine, and be of absorbing interest to every physician and person interested in this topic. A large edition will be issued and extra copies will be mailed to any address on the receipt of 75 cents in stamps or currency. Address, T. D. Crothers, M.D., Editor, Hartford, Conn.

---

WILLIAM M. WARREN, ESQ., medical publisher, Detroit, Mich., announces that the editor of the *Therapeutic Gazette*, Dr. Hobart A. Hare, has removed his office from 222 South 15th Street, to the N. W. Cor. 18th and Spruce Streets, Philadelphia.

---

MESSRS. BATTLE & CO., St. Louis, have issued chart No. 4 of the series, illustrating fractures of the long bones. This chart delineates fracture of the neck of the femur, with the muscles in color, as in the previous issues.

